# Bacteriorhodopsin–ZnO hybrid as a potential sensing element for low-temperature detection of ethanol vapour

**DOI:** 10.3762/bjnano.7.44

**Published:** 2016-04-04

**Authors:** Saurav Kumar, Sudeshna Bagchi, Senthil Prasad, Anupma Sharma, Ritesh Kumar, Rishemjit Kaur, Jagvir Singh, Amol P Bhondekar

**Affiliations:** 1 CSIR-Central Scientific Instruments Organisation, Sector 30C, Chandigarh 160030, India; 2 Academy of Scientific and Innovative Research, Rafi Marg, New Delhi 110011, India; 3 CSIR- Institute of Microbial Technology, Sector 39A, Chandigarh 160036, India,; 4 Research Services, University of Alberta, Edmonton, AB, Canada T6G2E1

**Keywords:** amphipol, bacteriorhodopsin, bio-hybrid, gas sensing, ITO, ZnO nanostructure

## Abstract

Zinc oxide (ZnO) and bacteriorhodopsin (bR) hybrid nanostructures were fabricated by immobilizing bR on ZnO thin films and ZnO nanorods. The morphological and spectroscopic analysis of the hybrid structures confirmed the ZnO thin film/nanorod growth and functional properties of bR. The photoactivity results of the bR protein further corroborated the sustainability of its charge transport property and biological activity. When exposed to ethanol vapour (reducing gas) at low temperature (70 °C), the fabricated sensing elements showed a significant increase in resistivity, as opposed to the conventional n-type behaviour of bare ZnO nanostructures. This work opens up avenues towards the fabrication of low temperature, photoactivated, nanomaterial–biomolecule hybrid gas sensors.

## Introduction

Nanomaterial–biomolecule conjugates have emerged into one of the most rapidly developing and sought after areas in modern biomolecular device fabrication and sensor design [1–4]. Novel approaches towards sensor design that employ biological material as the active element or associated active element have been widely explored [5–6]. Over the last few decades, the research in this domain has been directed towards the bio-inspired, self-assembly of monolayer/multilayer of thin films, biosensors, and protein-based photonic devices [7–9].

The application of proteins for enhancement in signal transduction has been demonstrated by a number of researchers [1,10]. In general, the major drawbacks associated with proteins are their low stability, poor retention of functional properties ex vivo, and a lack of immobilization techniques to prevent denaturation [4,8]. Interestingly, the protein bacteriorhodopsin (bR) has been proven to have significant stability against thermal, chemical and photochemical degradation [11–13]. Also, bR maintains its biological activity after immobilization on solid supports and exhibits charge transport in thin films [8,14–15]. These properties have attracted researchers for the development of novel bio-hybrid devices [5,7,16–19]. Hybrids of bR protein with various metal/metal oxides (e.g., Au, Ag, TiO_2_, SiO_2_) and polymers (e.g., PVA, gelatine) have been explored for photo–energy conversion, energy storage devices and gas sensing based on photo-conductive activity [12,14,20–22].

In parallel, ZnO and its hybrids have evolved as promising structures for sensing and semiconductor applications [23–24]. The preference of ZnO in applications for the formation of hybrid structures is due to its high band gap (3.39 eV), large excitonic binding energy (60 meV), and high isoelectric point (9.2) [23,25–27]. One of the early reported works by Heiland in 1959 demonstrated the use of ZnO as a gas sensing material [28]. Further, the advancement in concepts and techniques in nanotechnology resulted in the demonstration of the gas sensing capabilities of ZnO-based nanostructures [29–30].

Recently, researchers have explored innovative hybrid nanostructures based on the interaction of organic and inorganic materials in order to overcome the intrinsic limitations of ZnO (i.e., poor selectivity and high working temperature) [31–33]. Metal/metal oxide–bR hybrids were previously reported for bio-optoelectronic and solar cell applications [7,20]. However, a hybrid structure employing ZnO and bR protein has not been explored yet for gas sensing applications to the best of our knowledge. This work explores the possibility of overcoming the intrinsic limitations of ZnO, in particular the high operating temperature, by creating a ZnO/bR hybrid structure that exploits the charge transfer mechanism of bR.

## Results

In this work, ZnO thin films (ZnO-TF) and ZnO nanorods (ZnO-NRs) were grown via the hydrothermal method on indium tin oxide (ITO) substrates (25 × 25 mm) and both structures were used for the preparation of a sensitive film for gas testing. The precursor solution (zinc acetate dihydrate in 2-propanol reduced with monoethanolamine) was prepared using the sol–gel method and was spin-coated, layer-by-layer, to form the ZnO-TF. Further, the ZnO-TF was annealed (400 °C) for an hour in order to obtain a uniform size distribution of the ZnO grains [34]. The resulting average thickness and resistance of this film were observed to be 275 ± 10 nm and 2.22 × 10^2^ Ω, respectively. The film was further utilized for two purposes: to form ZnO-TF/bR and ZnO-TF/ZnO-NR/bR hybrid nanostructures. The synthesis of ZnO-NRs was carried out by suspending the seed layer (ZnO-TF) upside down in the growth solution consisting of zinc nitrate and hexamine. This inverted growth scheme was preferred in order to avoid contamination effects due to sedimentation and to achieve a uniform growth pattern [35–36]. Further, the suspension of wild-type, photoactive bR was prepared with aqueous amphipol (A8-35) in a 1:5 ratio in order to retain the conformational stability of bR in the solution phase without affecting its photoactive properties [37]. The drop casting method was used for the deposition of this suspension on the ZnO-TF as well as ZnO-NRs [14]. This modification serves to enhance the surface activity, which in turn enables the charge transfer in ZnO-TF/bR and ZnO-NR/bR interfaces [10,14].

**Figure 1:**
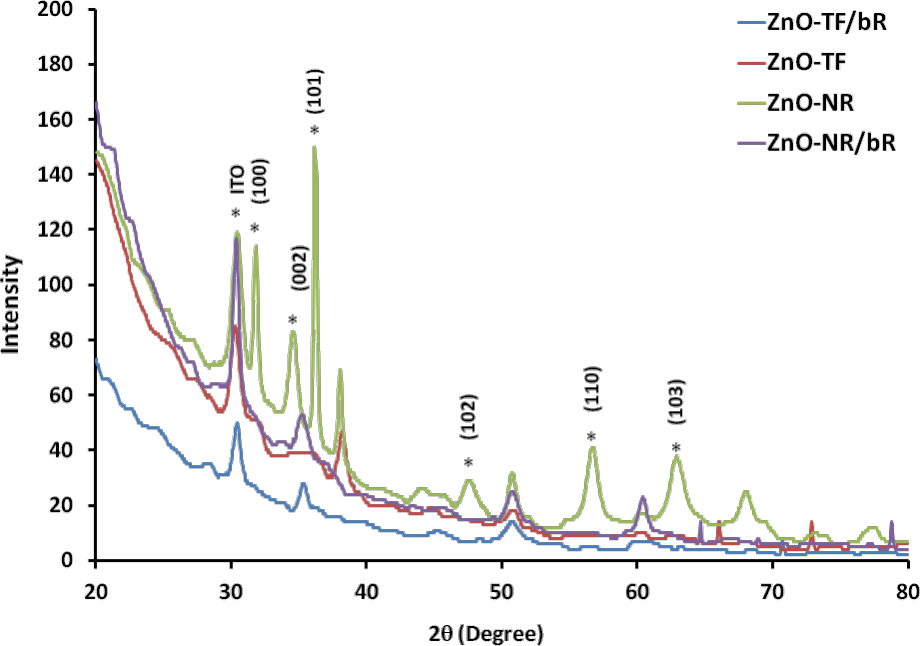
Comparative XRD patterns of the ZnO-TF, ZnO-TF/bR, ZnO-NR and ZnO-NR/bR structures.

In order to characterize the morphological changes, the surface of the hybrid structures was investigated by X-ray diffraction (XRD) and scanning electron microscopy (SEM). Figure 1 shows the XRD patterns of the ZnO-TF and ZnO-NRs on an ITO substrate, confirming the presence of ZnO. The strong peaks are in good agreement with the characteristic peaks of ZnO (marked with asterisk). A sharp peak at 34.4° indicates the preferential growth of the ZnO-NRs along the *c*-axis, normal to the substrate corresponding to the lattice plane (002) (JCPDS card number 36-1451) [38–39]. The average ZnO crystallite size on the ZnO-TF (as calculated by Scherrer’s equation) is 30 nm [40]. Further, the presence of bR on both the structures (synthesized under similar conditions) is supported by the significant difference in the intensity values of the XRD peaks with and without bR protein.

**Figure 2:**
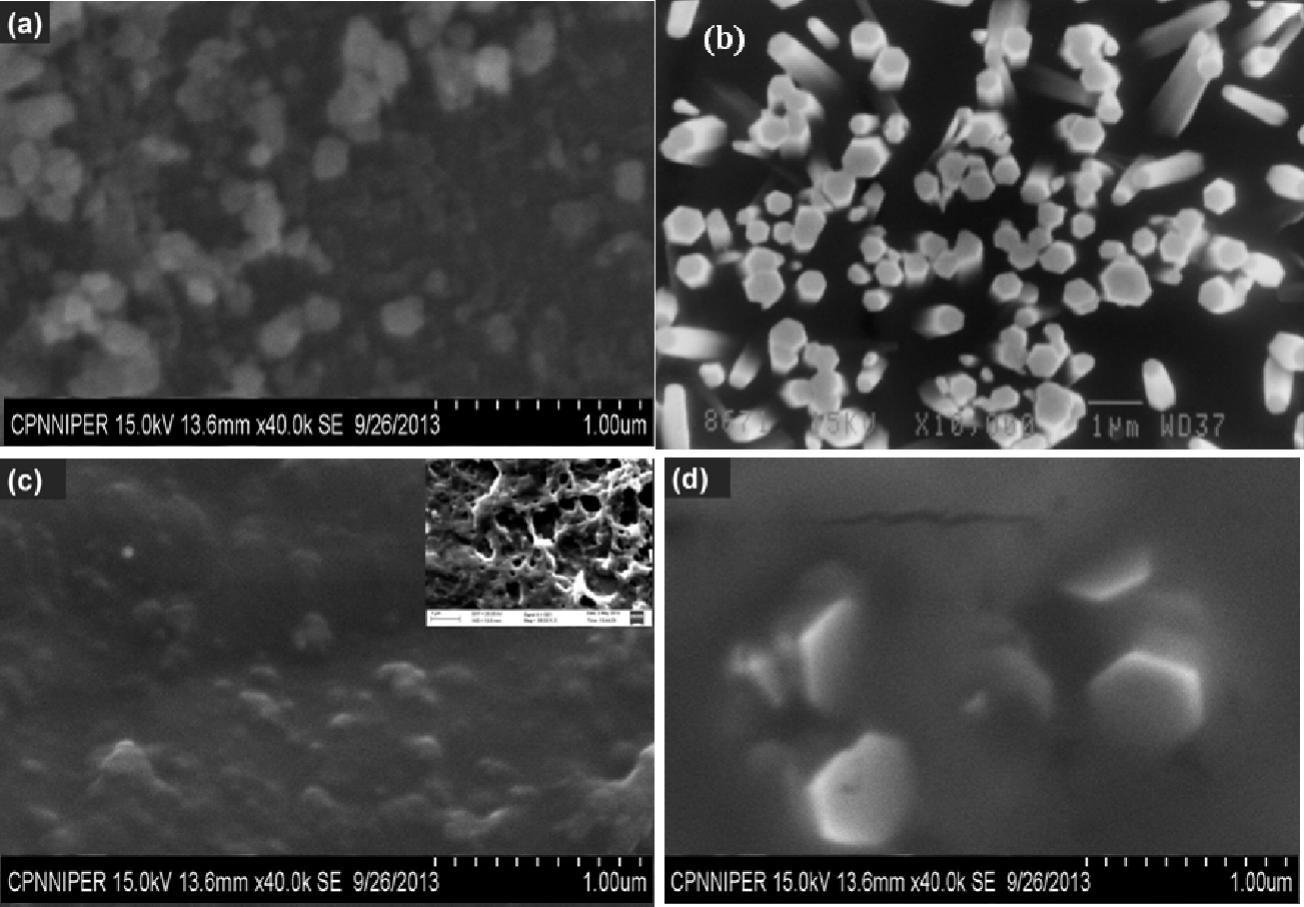
SEM characterization. (a) ZnO-TF has grown over the ITO substrate and was annealed at 400 °C. (b) ZnO-NR structure grown over the ZnO-TF seed layer. (c) ZnO-TF/bR hybrid structure prepared by drop casting. (d) ZnO-NR/bR hybrid structure.

Figure 2a–d shows SEM images of ZnO-TF, ZnO-NR on an ITO substrate and their respective hybrid structures with bR. The diameter of the hexagonal ZnO particles was found to be between 90–110 nm (average size 70 nm) in the Figure 2a. It can be observed from Figure 2b that the NRs are uniformly distributed and likely perpendicular to the substrate surface. The image shows the vertical growth of the rods with length 900–1300 nm and diameter 90–110 nm. The morphology of the hybrid structures (ZnO-TF/bR and ZnO-NR/bR) was distinctly different from nonhybrid structures, as is evident from Figure 2c,d.

**Figure 3:**
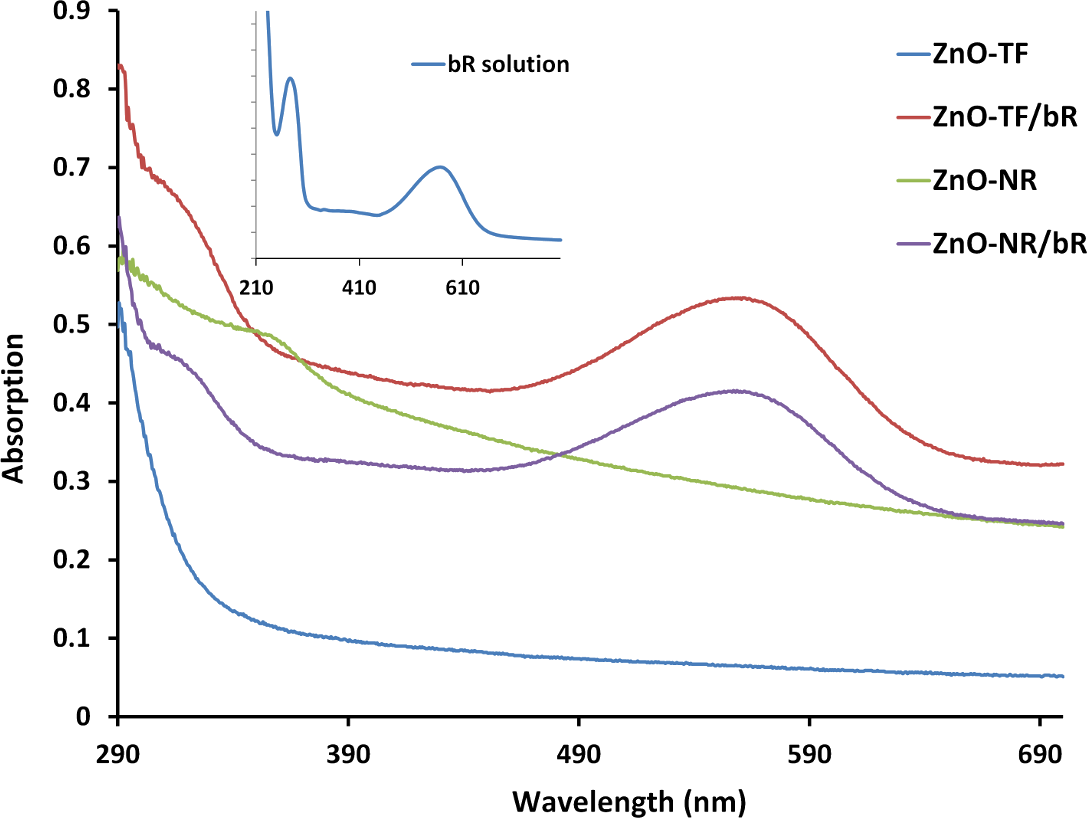
UV–vis spectra of ZnO-TF (blue); ZnO-NR/bR (purple); ZnO-NR (green) and ZnO-TF/bR (red) structures. The absorption spectrum of the bR protein (inset) indicates two specific absorption peaks of photoactive bR protein at 570 nm and 270 nm (in a 1 to 2 intensity ratio).

The structural and optical properties of ZnO-TF, ZnO-NR and their hybrid structures were assessed using UV–vis spectroscopy, photoluminescence spectroscopy (PL), Raman spectroscopy and Fourier transform infrared spectroscopy (FTIR). Figure 3 shows the absorbance spectra of ZnO-TF, ZnO-NR, bR solution in amphipol (bR/A8-35, inset figure) and the hybrid nanostructures. The UV–vis spectra of ZnO-TF and ZnO-NR show absorption near the band edge in the exciton absorption region at 372 nm and 347 nm, respectively [41]. These band edge absorptions have a similar trend in the respective hybrid structures, 362 nm for ZnO-TF/bR and 358 nm for ZnO-NR/bR. The blue shift in the excitonic absorption may be due to the reduction of grain size and improved structural quality of the surface. It is an indication of the increase in the band gap energy as the result of crystallization and strain factor [42–43]. The hybrid structures, ZnO-TF/bR and ZnO-NR/bR, show two specific characteristic peaks at 270 nm and 570 nm (inset figure), respectively. These are due to the superposition of the bands attributed to the aromatic residue of bR (including 8-tryptophan and 11- tyrosine) [44] and the π–π* transition in the retinal chromophore between two different energy levels upon exposure to the light [45–46]. However, the characteristic absorbance of bR due to retinal absorption at 570 nm was blue-shifted by 5 nm in ZnO-TF/bR and ZnO-NR/bR hybrids, which may be due to the interaction between the permanent dipole of retinal and charge layer of ZnO [46–47].

**Figure 4:**
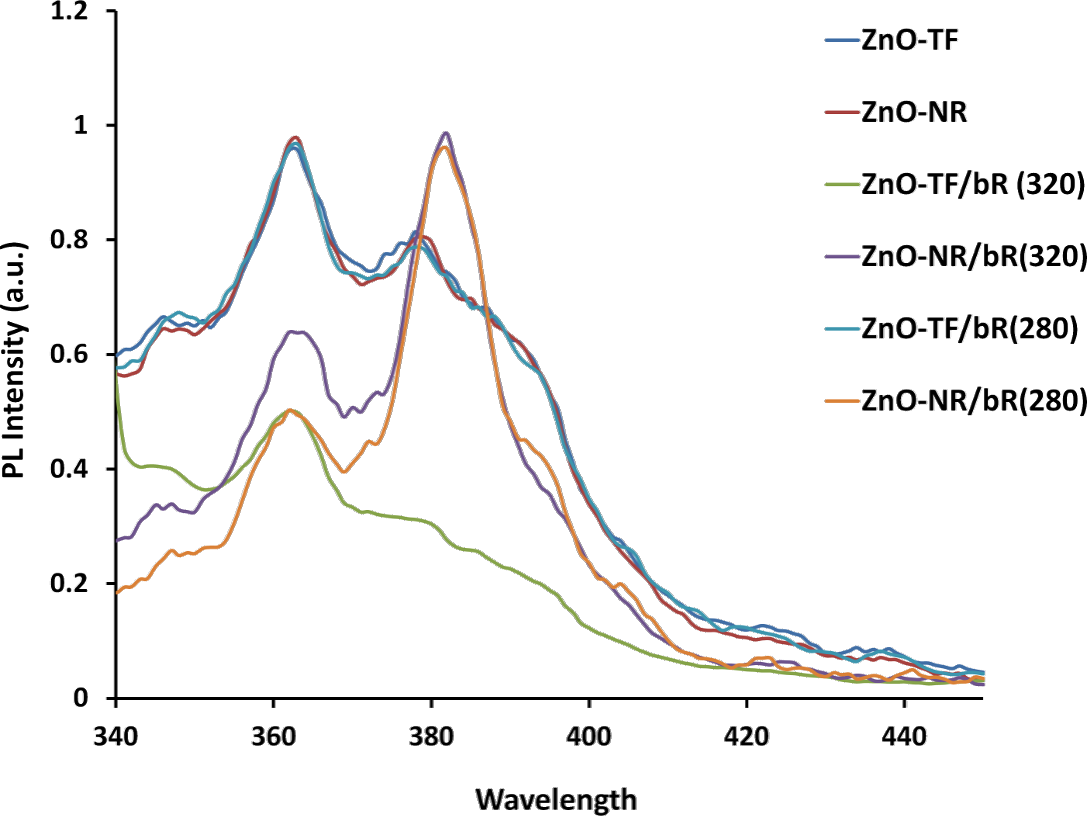
Photoluminescence spectral analysis for the ITO/ZnO-TF and ITO/ZnO-NR structures, and the respective hybrid structure for the structural analysis of the surface.

Figure 4 illustrates the PL spectrum of ZnO-TF and ZnO-NR (at an excitation wavelength of 320 nm) and their hybrid structures (at an excitation wavelength of 320 nm and 280 nm). The strong emission band close to 368 nm is the band edge emission, and the emission around 383 nm may be attributed to the recombination of free excitons through an exciton–exciton collision process [48–49]. Also, the peak positions were almost same in ZnO-TF and ZnO-TF/bR, whereas a red shift of 6 nm was observed in ZnO-NR and ZnO-NR/bR. This red shift may be due to the microcavity effect of ZnO-NRs and its excitonic recombination with the emission of bR protein [50]. Visible band emissions were not prominent in any of the structures, suggesting the absence of structural defects and impurities [51–52], as well as higher crystallization and less oxygen vacancies [53]. As observed from Figure 4, the PL intensity increases with increasing excitation wavelength (i.e., 280 nm and 320 nm), which may be attributed to electron–hole plasma recombination shift by band renormalization [54].

**Figure 5:**
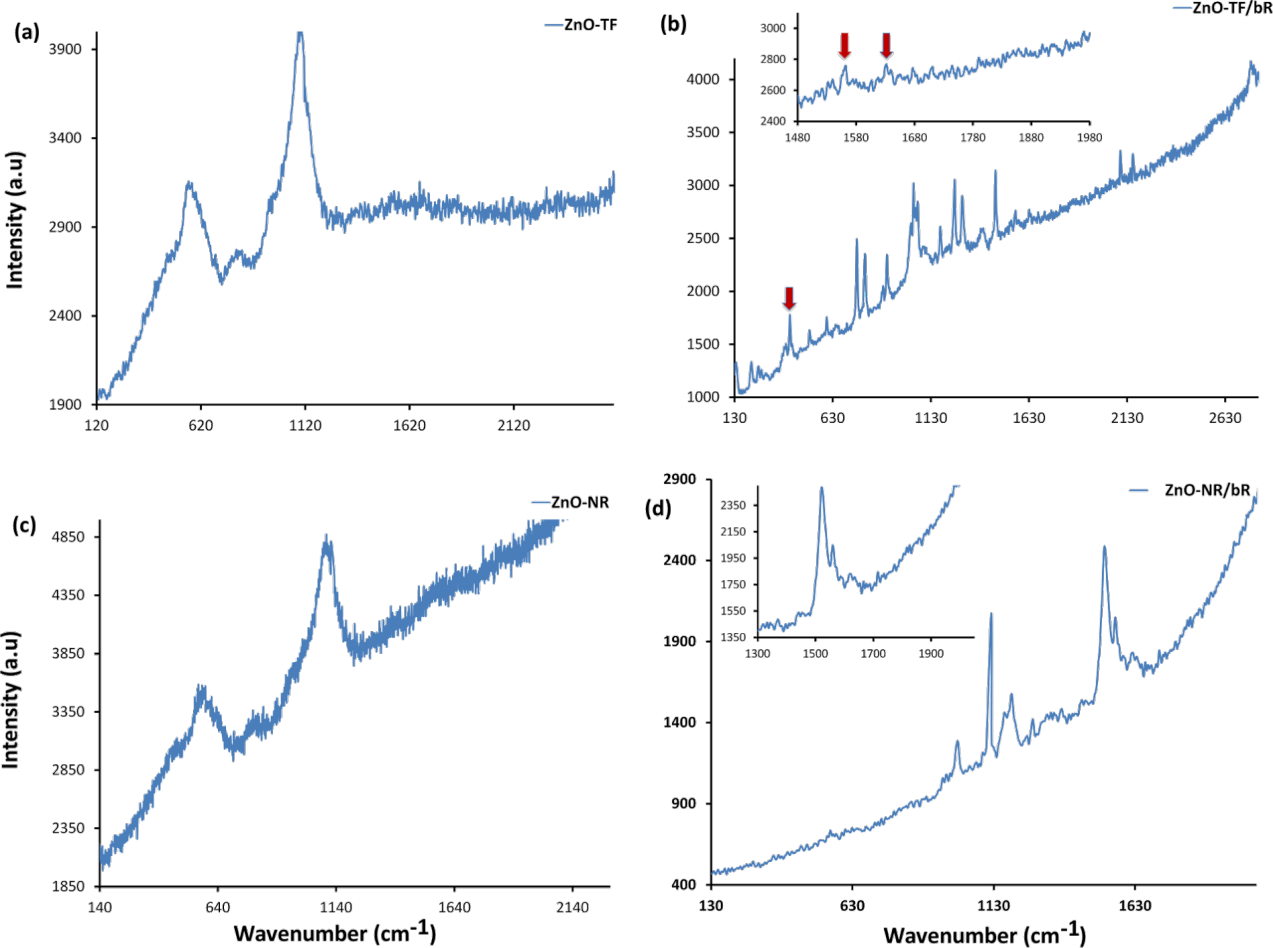
Raman spectra. (a) ZnO-TF shows the characteristic peaks for ZnO at 467 cm^−1^, 563 cm^−1^ and 800 cm^−1^; (b) ZnO-TF/bR hybrid structure showing characteristic peaks of both ZnO and bR, indicating the preservation of the functional properties of the protein. Inset image shows amide I and amide II peaks of bR protein. (c) ZnO-NR structure illustrates the major ZnO vibrational peaks at 476 cm^−1^, 570 cm^−1^, 806 cm^−1^ and 1106 cm^−1^. (d) ZnO-NR/bR hybrid structure. Inset image shows specific amide I and amide II peak of bR protein.

Figure 5 shows the Raman spectra for all the structures at 514 nm excitation wavelength. ZnO has wurtzite symmetry with the C_6_*_v_* point group. Group theory predicts that there is an A_1_ branch, a doubly degenerate E_1_ branch, two doubly degenerate E_2_ branches, and two B branches. The A_1_, E_1_ and E_2_ modes are Raman active; the B modes are inactive [55]. It can be observed from Figure 5a that the fundamental optical mode vibrations are at 437 cm^−1^ and 583 cm^−1^ corresponding to the E_2_ mode and A_1_(LO) mode, respectively, in ZnO-TF [56–57]. The E_2_ mode is associated with the vibration of the heavy Zn sublattice of crystalline wurzite ZnO, and the A_1_(LO) mode is associated with intrinsic lattice defects [58–59]. The Raman spectra of ZnO-NR in Figure 5c shows a shift in the A_1_(LO) mode vibration by 8 cm^−1^, indicating the possibility of a confinement effect [60]. The vibrational spectra of the hybrid structures (Figure 5b,d) shows characteristic peaks of bR protein at 1528 cm^−1^ (C=C stretching) and fairly strong fingerprint spectra in the range of 1150–1220 cm^−1^ (C–C stretching) [61]. However, there is a small shift in the characteristic vibrational modes of bR, which may be due to the electrostatic interaction between the ZnO and bR protein [62].

**Figure 6:**
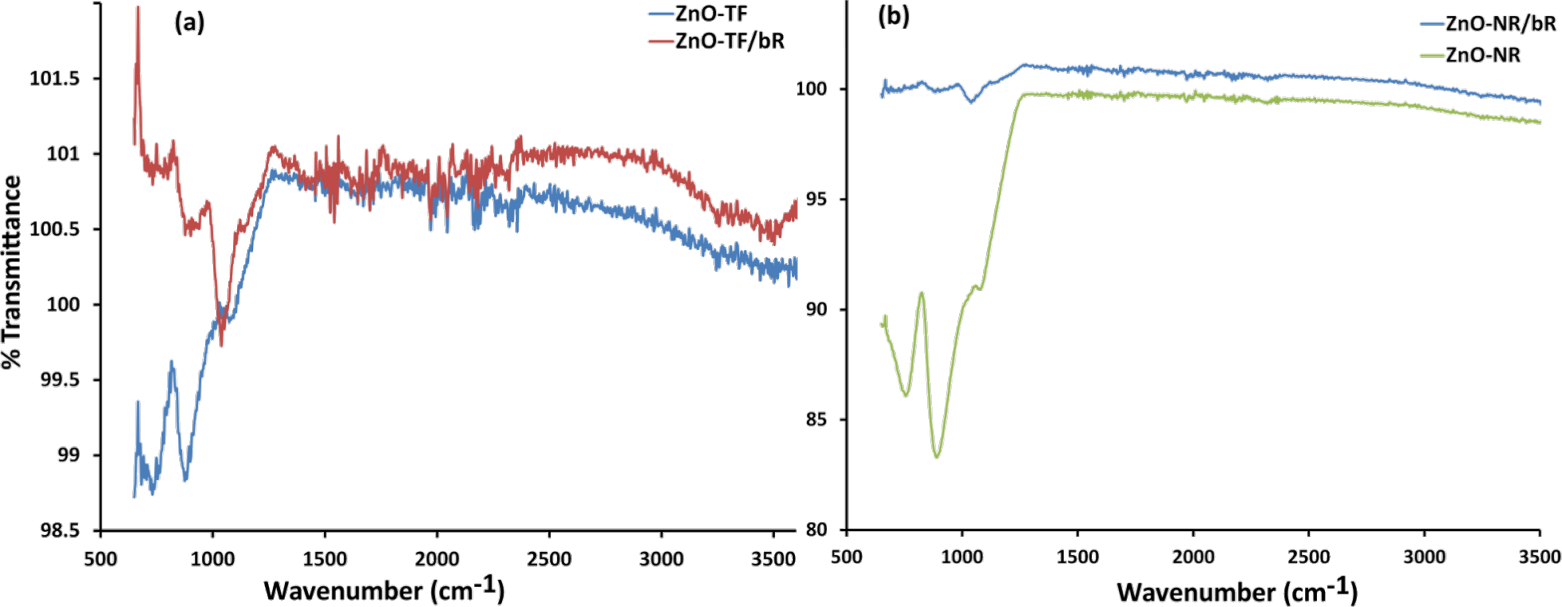
FTIR spectral analysis. (a) ZnO-TF and ZnO-NR/bR; (b) ZnO-NR and ZnO-NR/bR. The vibrational spectra of the ZnO-TF and ZnO-NR structures show the characteristic peaks of ZnO with a red shift (≈10 cm^−1^) in the ZnO/NR structure, indicating the possibility of a confinement effect. The characteristic vibrational modes of bR were also observed in the hybrid structures with a small red shift.

Figure 6 shows the FTIR spectra for the ZnO-TF and ZnO-NR structures, and their respective hybrid structures (ZnO-TF/bR and ZnO-NR/bR). Spectral analysis of the hybrid structures shows a shift in the frequency of the characteristic peaks of bR protein [63–67], which might be due to the electrostatic interaction between the bR protein and the ZnO nanostructures [68–69]. The peak at 1040 cm^−1^ (region 880–1050 cm^−1^) shows the hydrogen out-of-plane (HOOP) vibrational mode, which indicates a twist in the skeleton of the protein. The comparatively weak feature within 1150–1500 cm^−1^ may be attributed to C–O–C asymmetric stretching, O–H bending, C=C stretching and C–H bending of the retinal chromophore, amino acids and lipids [66]. Further investigation indicates a small change in the amide I vibration spectra (at 1660 cm^−1^) due to the C=O stretch in the skeleton and amide II vibration spectra (at 1540 cm^−1^) due to N–H in-plane bending, which reflects the secondary structure of the protein (β-structure) [70–72]. Higher frequencies at 3070, 2950, 2920 and 2850 cm^−1^ are attributed to the alkyl C–H stretching vibration of both lipid and protein amino acid [63,65,73].

**Figure 7:**
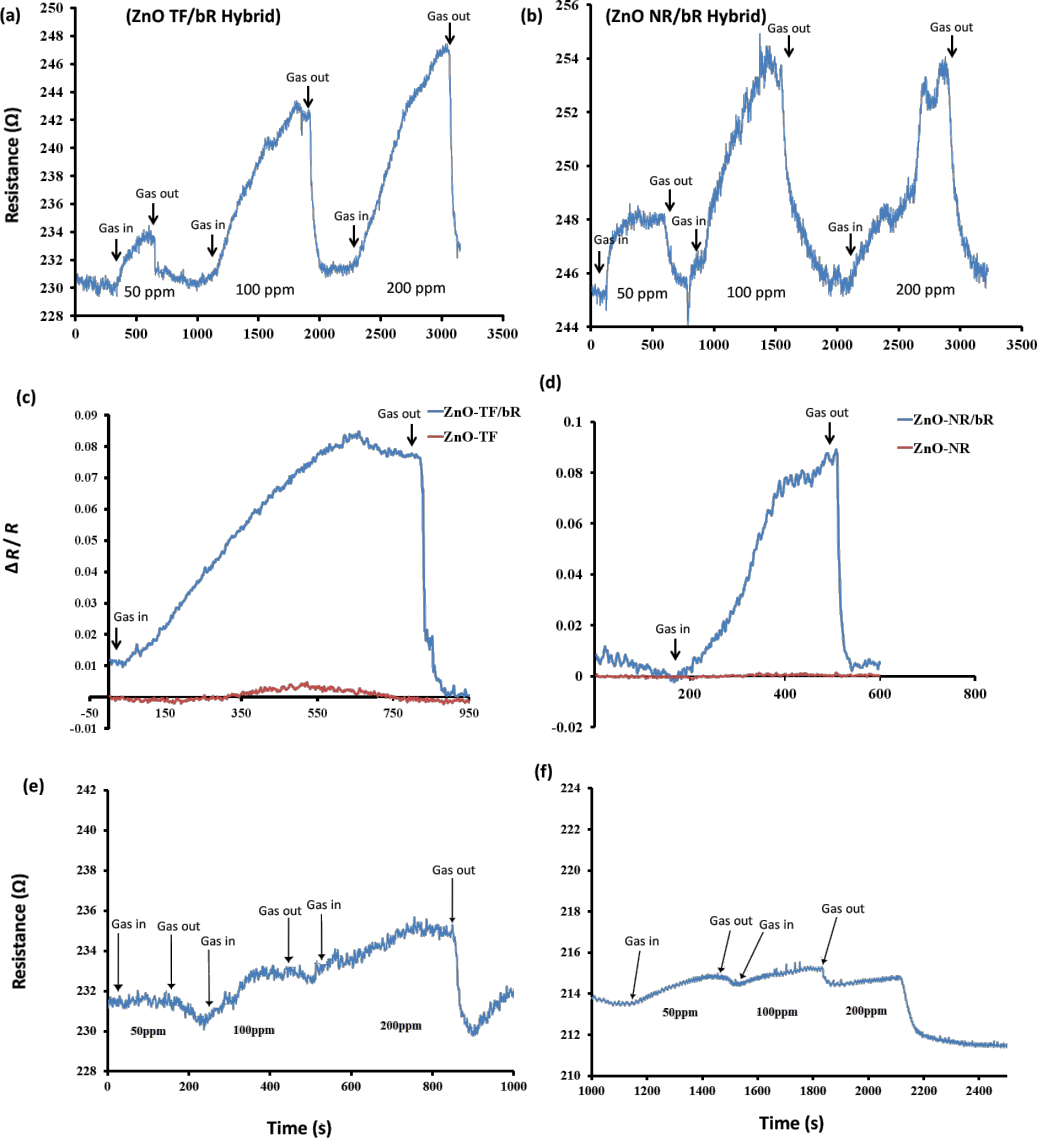
Dynamic response of the ethanol vapour sensing tests at 70 °C for 50, 100 and 200 ppm concentrations for (a) ZnO-TF/bR and (b) ZnO-NR/bR structures. Comparative dynamic response diagram of hybrid and nonhybrid ZnO structures at 70 °C for 100 ppm concentration for (c) ZnO-TF and ZnO-TF/bR, and (d) ZnO-NR and ZnO-NR/bR. A similar response at 65 °C for the 50, 100 and 200 ppm concentrations is shown for (e) ZnO-TF/bR and (f) ZnO-NR/bR.

The gas sensing tests were carried at 55, 65 and 70 °C for 50, 100 and 200 ppm concentrations of ethanol gas under ambient light conditions using a standard Taguchi gas sensing kit [74]. It has been widely reported that bR denatures between 75 to 100 °C, depending upon its phase, morphology, experimental conditions, etc. In order to protect our sample, excessive temperatures were avoided, and the experiments were restricted to 70 °C. A noticeable increase in the resistance of the ZnO-TF/bR and ZnO-NR/bR structures was observed upon exposure to ethanol gas of 50 ppm, 100 ppm, and 200 ppm concentrations. Figure 7a,b shows the response of the ZnO-TF/bR and ZnO-NR/bR structures for the consecutive concentration increase (50 ppm, 100 ppm, 200 ppm) at 70 °C. The response time for the ZnO-TF/bR and ZnO-NR/bR structures was observed to be 11 and 4.3 min, respectively, and the recovery time was 1.7 and 1.5 min, respectively. The experiments were repeated to ensure stability of the response of the hybrid structures, wherein no significant difference was observed. This confirmed that the deposited bR did not denature. Figure 7c shows the response of the ZnO-TF and ZnO-TF/bR structures at 70 °C for 100 ppm concentration, and Figure 7d shows the response of the ZnO-NR and ZnO-NR/bR structures at 70 °C for 100 ppm concentration. In both cases the enhancement in sensitivity of the hybrid structures is noticeable. Figure 7e,f shows the poor response of the hybrid structures at 65 °C.

The n-type gas sensing behaviour of ZnO at very high temperature (≥300 °C) is accompanied by the dehydration/dehydrogenation reaction of ethanol, releasing CO_2_ and H_2_O [75]. However, contrary to this, both the hybrid structures in our study showed p-type behaviour. This observed inconsistency is due to the increase in barrier height of approximately 2.90 meV for the ZnO-NR and 2.10 meV for the ZnO-TF hybrid structure (measured from the work function equation), thus contributing to the overall increase in resistance [76]. One of the possible explanations can be the dipole–dipole interactions between the extracellular site of the protein and ethanol molecules, which leads to the electron transfer between the surface and the interior layer [77–78]. Secondly, the direct adsorption of ethanol molecules may cause changes in the morphology of protein molecules, resulting in an increase in the surface resistance [77]. The formation of localized heterojunctions at the ZnO/bR nanostructure, and the possibility of exchanging charge carriers at the hybrid interface, are expected to strongly affect the electronic properties of the resulting nano-hybrid material. The hybrid ZnO-TF/bR and ZnO-NR/bR structures form the main percolative network through which charge carriers can move [79–80].

## Conclusion

Nanomaterial–biomolecule hybrids based on ZnO-TF or ZnO-NR functionalized with bR protein have been successfully fabricated on ITO substrates. The morphological, optical, and electrical characterization of these devices was presented. The behaviour of ZnO-TF/bR and ZnO-NR/bR hybrids has been studied upon exposure to ethanol vapour at 50, 100 and 200 ppm concentrations at 55, 65 and 70 °C. The sensing results for both the nano-hybrid structures showed good response towards ethanol gas at 70 °C. Typically, ZnO shows n-type behaviour upon exposure to reducing gases like ethanol, whereas p-type behaviour has been observed in the hybrids. This may be attributed to the heterojuction of ZnO/bR, which provides a percolative network for the movement of charge carrier. This work demands further investigation related to device characteristics (e.g., specificity, sensitivity, photoactivity, concentration variability, threshold limit) and device fabrication with more precision with application specificity.

## Experimental

### ZnO thin film (ZnO-TF) preparation

ZnO nanoparticles were synthesized by hydrothermal growth [81–82]. Zinc acetate dihydrate (0.1 M) was dissolved in 20 mL of propanol. This solution was stirred at 40–60 °C for 5–10 min. Then, monoethanolamine (0.1 M) was poured drop-by-drop into the solution. A clear transparent solution so obtained was stirred at 500 rpm for 2 h. This solution was kept in dark for the next 12 h and used for coating. Spin coating (Millman, single-stage coating unit) was performed at 3000 rpm for 20 s to obtain a thin film of ZnO nanoparticles.

### ZnO nanorod (ZnO-NR) synthesis

ZnO-NRs were grown on the ZnO-TF substrate by the hydrothermal method [36,83]. Zinc nitrate hexahydrate (0.2 M) was used as a precursor salt and was dissolved in aqueous ethanol (30%). A separate equimolar solution of hexamethylenetetramine (HMT) was also prepared in aqueous ethanol (30%). These solutions were stirred for 30 min at 60 °C. The growth solution was obtained by slow titration of HMT solution against zinc nitrate solution [34]. The ZnO substrate was kept inverted in this growth solution for 3 h at 80 °C undisturbed. The substrate was removed from the growth solution and dried at room temperature. Then, this substrate was washed with DI water and dried in a vacuum desiccator.

### Hybrid nanostructure (ZnO-TF/bR and ZnO-NR/bR) synthesis

Amphipol (APol) A8-35, an amphiphilic polymer, was used to stabilise the protein. The bR protein suspension was prepared in aqueous A8-35 (10%) solution in the ratio of 5:1 (w/w). The bR protein suspension was stored under dark conditions at 4 °C [37]. Finally, hybrid nanostructures (ZnO-TF/bR and ZnO-NR/bR) were fabricated by manually drop casting 50 µL of the bR protein suspension (using a micropipette) on the ZnO-TF and ZnO-NR substrates (625 mm^2^ area) [14]. The deposited film was dried in vacuum at room temperature by using a standard 3 L desiccator connected to a Millipore vacuum pump (4 bar).

### Structural characterisation

X-ray diffraction patterns for the ZnO-TF, ZnO-NR and respective hybrid structures were recorded using an X-ray diffractometer (Rikgo) with Cu Kα radiation of wavelength λ = 0.1541 nm in the scan range 2θ = 20–80°. The surface morphology of these samples was investigated using scanning electron microscope (SEM, Hitachi) with an accelerating voltage of 5–15 kV. The film thickness was measured using a surface profilometer in contact mode (Taly-surf PGI 120).

### Raman and optical spectral analysis

The optical absorption spectra of all the nanostructures were recorded using a UV–vis spectrophotometer (Hitachi, 3900 H). The photoluminescence (PL) spectrum was measured using a Varian spectrofluorometer. The Raman spectra were taken using a 514 nm wavelength laser source with a Renishaw Invia spectrophotometer. A Perkin Elmer spectrophotometer was used for the FTIR analysis of the samples. The FTIR analysis was carried in ATR mode and the transmission spectra were analysed.

### Gas sensing setup

**Figure 8:**
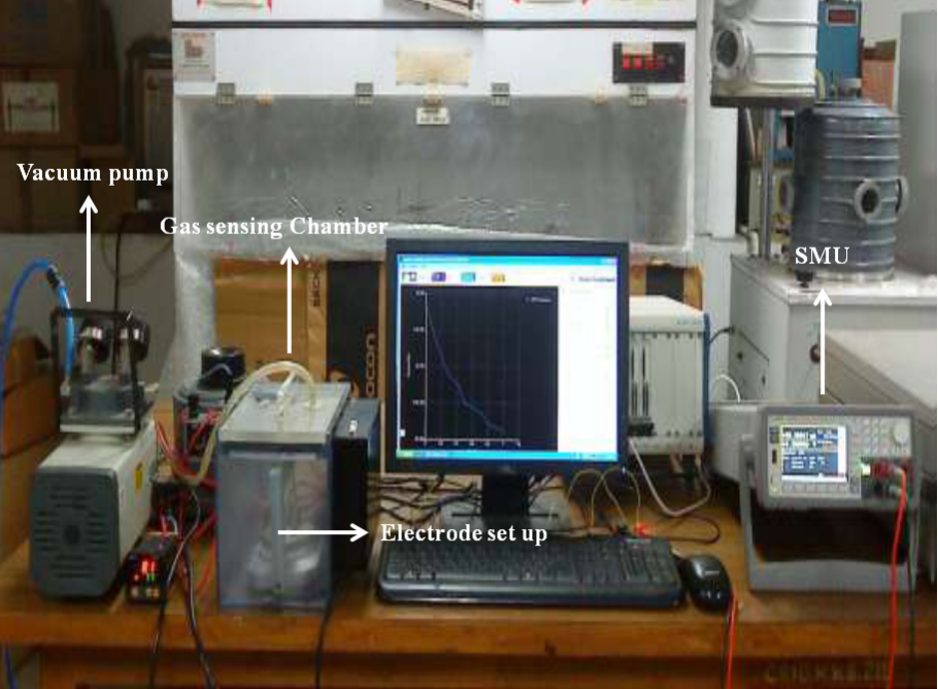
Gas sensing setup.

The gas sensing measurements were carried out in a customized chamber (Taguchi Gas sensing kit). The chamber was sealed to minimize the effect of atmosphere on the substrate. The ZnO-TF and ZnO-NR nanostructures, with and without bR (nonhybrid surface), were placed on a temperature-controlled heating plate inside the microreactor chamber. The chamber was controlled by a PID temperature controller (Schnieder Electric, Japan). The resistance across the substrate was measured using spring-loaded copper electrodes (contact points lightly covered with silver paste and spaced 10 mm apart) connected to an Agilent Precision source/Measure Unit Model B2901. The baseline resistance (i.e., without gas) of ZnO-TF, ZnO-NR, ZnO-TF/bR and ZnO-NR/bR structures was observed to be 2.22 × 10^2^ Ω, 1.45 × 10^2^ Ω, 2.31 × 10^2^ Ω and 2.45 × 10^2^ Ω, respectively. The sensing setup is shown in Figure 8. Known volumes of ethanol were injected onto a petri dish mounted on a preheated (80 °C) heating plate placed inside the chamber. The vapour concentration was calculated using the equation given by Yinhua et al. [74]:

[1]
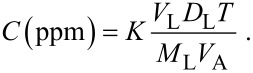


where, *K* is a constant having a value of 8.2 × 10^4^, *T* is the operating temperature in Kelvin, *V*_A_ is the volume (mL) of the diluting gas (which is equal to the volume of the test chamber), and *V*_L_, *D*_L_ and *M*_L_ refer to the volume (mL), density (g mL^−1^) and molecular weight (g mol^−1^) of the liquid organic analyte (ethanol), respectively.
